# Phase II trial of cisplatin and capecitabine in patients with squamous cell carcinoma of the head and neck, and correlative study of angiogenic factors

**DOI:** 10.1038/sj.bjc.6602275

**Published:** 2004-12-14

**Authors:** R Hitt, A Jimeno, M Rodríguez-Pinilla, J L Rodríguez-Peralto, J M Millán, A López-Martín, A Brandariz, C Peña, H Cortés-Funes

**Affiliations:** 1 Medical Oncology Department, University Hospital 12 de Octubre, Madrid, Spain; 2 Pathology Department, University Hospital 12 de Octubre, Madrid, Spain; 3 Radiology Department, University Hospital 12 de Octubre, Madrid, Spain; 4 Otorrhinolaringology Department, University Hospital 12 de Octubre, Madrid, Spain; 5 Radiotherapy Department, University Hospital 12 de Octubre, Madrid, Spain

**Keywords:** cisplatin, capecitabine, head and neck squamous cell carcinoma

## Abstract

The combination of cisplatin and capecitabine was evaluated in patients with recurrent or unresectable squamous cell carcinoma of the head and neck (HNSCC), and outcome parameters were correlated with the expression of thymidine phosphorylase (TP), thymidilate syntetase (TS), vascular endothelial growth factor receptor (VEGFR) 1–3, and microvessel density (MVD). Patients with recurrent or unresectable HNSCC were eligible if they had received prior neoadjuvant chemotherapy, concurrent chemo-radiotherapy, or no prior systemic therapy. Patients received cisplatin (75 mg m^−2^ day 1), and capecitabine (2000 mg m^−2^ day 1–14) every 3 weeks. A total of 41 patients received 194 cycles. In all, 16 complete responses (39%) and 12 partial responses (29%) were documented, for an overall response rate of 68% (95% CI, 53–80%). Grade 3–4 uncomplicated neutropenia was documented in five subjects. Asthenia, anorexia, hand–foot syndrome, and constipation were the most frequent nonhaematologic events. Median progression-free and overall survival were 6.4 and 12.6 months. Cytoplasmic TP expression was more prevalent in patients with a laryngeal location *vs* other, and in patients with a recurrence *vs* primary disease. Microvessel density count was higher in patients with recurrent *vs* primary disease. The combination of cisplatin and capecitabine is effective in recurrent or unresectable HNSCC, and shows a manageable toxicity.

Head and neck squamous cell carcinoma (HNSCC) accounts for most malignancies that arise in the head and neck region (Liggett and Forastiere, 1995). Two-thirds of the patients present with advanced locoregional disease, and despite combined modality approaches with chemotherapy and radiotherapy or surgery, locoregional and distant failure occurs in up to 60 and 25% of the patients, respectively, and the 3-year survival rate remains below 30% (Dimery and Hong, 1993; Jacobs, 1994). The combination of cisplatin and infusional 5-fluorouracil (5FU) is a widely accepted schedule in the locally advanced or recurrent disease setting, with response rates of 60–80 and 30–40%, respectively; in the latter setting, median overall survival is 6 months (Campbell *et al*, 1987; Forastiere *et al*, 1992; Jacobs *et al*, 1992; Clavel *et al*, 1994).

Capecitabine (Xeloda™) is an oral fluoropyrimidine prodrug that is efficiently absorbed in the gastrointestinal tract and then transformed by a sequence of reactions to 5′-deoxy-5-fluorocytidine (5′DFCR), deoxyfluridine (5′DFUR), and finally to 5-fluorouracil (5FU) by thymidine phosphorylase (TP). Thymidine phosphorylase is preferentially expressed in malignant cells and is responsible for the higher intratumoral *vs* systemic 5FU concentrations found after capecitabine administration (Kono *et al*, 1983; Takebayashi *et al*, 1996). Capecitabine results in tissue to plasma 5FU concentration ratios higher than intravenous 5FU (Ishikawa *et al*, 1998a, 1998b). The synergistic activity found between cisplatin and 5FU prompted the evaluation of the combination of both agents in a phase I, disease-directed study in 21 patients with recurrent HNSCC; the recommended doses were cisplatin 100 mg m^−2^ on day one of a 21-day cycle, and capecitabine 1000 mg m^−2^ bid days 1–14 (Pivot *et al*, 2003).

Thymidine phosphorylase is a relevant target by being both an angiogenesis-promoting factor, and the intracellular activator of 5FU prodrugs. This enzyme is widely expressed in humans cancers (Zimmerman and Seidenberg, 1964), and higher levels of TP expression are associated with increased intratumoral microvessel density (MVD) and an unfavourable prognosis (Okamoto *et al*, 2001). Nuclear TP (TP-nuc) correlated with MVD, and was inversely related with response and outcome in HNSCC patients treated with 5FU, cisplatin and radiotherapy (Koukourakis *et al*, 2000). However, the relationship between these and other angiogenic-related factors, such as the expression of the vascular endothelial growth factor receptors (VEGFR) 1–3, and outcome parameters of HNSCC patients treated with cisplatin and capecitabine, has not been explored. The present study addresses these issues, while evaluating the feasibility and clinical activity of the combination of cisplatin and capecitabine in recurrent or unresectable HNSCC.

## PATIENTS AND METHODS

### Patient eligibility

Patients were required to have 18 years of age or older with histologically documented HNSCC that was recurrent or unresectable, measurable disease, Karnofsky performance status (KPS) ⩾70, life expectancy of 12 weeks or longer, and adequate bone marrow, hepatic and renal function (absolute neutrophil count (ANC) ⩾2.0 × 10^9^ l^−1^, platelet count ⩾100 × 10^9^ l^−1^, haemoglobin level ⩾10.0 g dl^−1^, AST or ALT levels of <1.5 × the upper limit of normal, alkaline phosphatase level <5 × the upper limit of normal, normal bilirubin level, and a creatinine clearance ⩾50 ml min^−1^). Patients could have received one prior regimen of chemotherapy as neoadjuvant, adjuvant or concurrent therapy with radiation, as long as it was terminated more than 6 months before disease recurrence. Patients with grade 2 or higher peripheral neuropathy or other serious medical or psychiatric condition were excluded. The scientific review board of our institution granted protocol approval. Patients were required to provide written informed consent prior to enrolment into the study. Within 3 weeks before study entry, a computed tomography (CT) scan of the head and neck was performed. Within 1 week of study entry, all patients had a complete clinical history and physical examination, complete blood counts, serum biochemistry tests (liver and renal function tests, and electrolytes), urinalysis, and electrocardiogram.

### Treatment plan

Treatment consisted of cisplatin (75 mg m^−2^ as a 30-min i.v. infusion on day 1), followed by capecitabine (1000 mg m^−2^ orally twice daily, on days 1–14), every 21 days. Standard mannitol and i.v. hydration accompanied cisplatin administration. Prophylactic antiemetics included i.v. ondansetron (8 mg) prior to chemotherapy, and oral ondansetron 4 mg three times daily for 2 days. Treatment was administered on an outpatient basis for a maximum of six cycles. Re-treatment on day 22 required an ANC count ⩾1.5 × 10^9^ l^−1^, a platelet count ⩾100 × 10^9^ l^−1^, a creatinine clearance rate >50 ml min^−1^, and resolution of all nonhaematological toxicities (except alopecia and fatigue) to baseline or less than grade 1. In case of a delay longer than 14 days, the patient was removed from the study. The doses of cisplatin and capecitabine were reduced following specific guidelines. Toxic events were recorded on a continuous basis, and followed until they were resolved to baseline or less than grade 1. Treatment compliance was evaluated using patient diaries and accounting unused drug. History and physical examination, complete blood cell counts, and serum biochemistry tests were performed at weekly intervals during treatment. Re-staging CT scans, chest radiographs, and upper respiratory tract examinations were performed every 3 cycles (9 weeks), or when clinically indicated.

### Study end points

The primary end point of the study was tumour response. The criteria used to define response were standard RECIST criteria (Therasse *et al*, 2000). Secondary efficacy parameters were progression-free survival (PFS) and overall survival (OS). Progression-free survival and OS were defined as the time from diagnosis of recurrent or advanced disease to the last contact, or progression or death, respectively. Adverse events were classified and graded according to the National Cancer Institute Common Toxicity Criteria version 3.0. Patients were considered evaluable for response and toxicity once therapy was initiated.

### Tissue microarray (TMA) design

A tissue arrayer device (Beecher Instrument, Silver Spring, MD, USA) was used to construct the TMAs. All slides were reviewed by expert pathologists (MRP and JLRP) to select areas containing tumour cells and to exclude those areas showing necrosis, inflammation, and queratinisation. To assess reproducibility, cylinders of 0.6 mm in diameter from two different areas were taken in each case. Tissue microarray blocks were constructed with the duplicated test sample and 12 controls. The latter specimens were used to ensure the quality, reproducibility, and homogenous staining of the slides, and consisted on archival reactive tonsil tissue. The samples were blindly scored, and when discordances between the duplicated specimens occurred an additional area was considered.

### Immunohistochemistry

Sections (3-*μ*m thick) were cut from the TMA and transferred to silanised glass slides. The sections were dried for 16 h at 56°C before being de-waxed in xylene and re-hydrated through a graded ethanol series and washed with phosphate-buffered saline. For antigen retrieval a treatment in a pressure cooker for 15 min in citrate buffer at 95°C was performed. Immunohistochemical staining was performed in an automated immunostainer, using the biotin–streptavidin-peroxidase procedure with diaminobenzidine as the chromogen. Haematoxilin was used for counterstaining. The following antigens were stained: TS (TS 106, Neomarkers, MS-471, 1 : 10 LSAB), thymidine phosphorylase (PD-ECGF Ab-1, Neomarkers, PGF.44C, prediluted during 30 min), VEGF-R1/ Flt-1 (h-225) (Polyclonal rabbit, Santa Cruz, 1/800), VEGFR2/FLK-1 (Monoclonal A-3, Santa Cruz Biotechnology, Inc., 1/50), VEGF-R1/FLT-1 (C-17) (Policlonal rabbit, Santa Cruz, 1/150). Reactivity for all antibodies was quantified by use of a visual grading system based on the intensity of cytoplasm staining (0–2) as follows: grade 0: no immunoreactivity, grade 1: if any specific staining, slightly stronger than background staining was detected, and grade 2: if clear immunoreactivity in more than half of the cancer cells were detected. The association of histologic factors with site of disease, type of disease, stage, and response to treatment was analysed; the impact of these factors on progression-free and overall survival was evaluated. Assessment included both no *vs* any expression, as well as no plus low *vs* high expression.

### Microvessel counting

Blood vessels were visualised by staining endothelial cells for CD31 (Mouse monoclonal antibody JC70, Dako, Prosan, Belgium). One section per tumour was analysed. The entire section was analysed at × 20–40 magnification to identify the areas of most intense neovascularisation, that were identified as having the highest density of brown staining. Vascular hotspots were suitable for analysis provided they were adjacent to tumour cells. Whenever such a highly vascularised area was encountered at × 20–40 magnification, individual microvessels were counted and expressed as microvessels per field (MPF) at × 200 magnification. Neither vessel lumens nor the presence of red blood cells was used to define a microvessel, and no exclusion criteria based on size were used. The mean of the five highest counts per tumour was taken for further analyses. The predefined cutoff value for categorical evaluation of MVD was the median of the MVD of the population studied.

### Statistical analysis

The trial followed a two-stage Simon Minimax design (Thall *et al*, 1989), allowing early closure in case of treatment failure. The null hypothesis of a true response rate of 50% or lower was evaluated against the alternative hypothesis of a true response rate of 70% or higher (alpha error 0.05, beta error 0.20). In all, 13 responses were required in the first 23 patients to continue accrual, and 24 responses out of 37 patients were required to consider the regimen promising. Confidence intervals (CIs; 95%) were calculated with the exact method. Survival was estimated by the Kaplan–Meier product limit method (Kaplan and Meier, 1958). A Cox proportional hazards model was created to assess prognostic or predictive factors in a multivariate fashion. All tests were two-sided at the 0.05 level of significance. The SPSS (version 10.0, Chicago, USA) package was used for statistical analyses.

## RESULTS

### Patients

Between November 2001 and November 2002, 41 patients with locally advanced HNSCC were accrued into the study. All patients were assessable for toxicity and survival, and response could be evaluated in 40 patients. Demographic and clinical characteristics of the subjects are summarised in Table 1

**Table 1:** Patient characteristics

	**Patients (*n*=41)**	
**Characteristic**	**No.**	**%**
Sex		
Male	39	95
Female	2	5
		
*Age (years)*		
Median	54	
Range	33–73	
		
Karnofsky performance status		
90	11	27
80	24	58
70	6	15
		
Initial staging at diagnosis		
II	4	10
III	5	12
IV	32	78
		
Primary tumour site		
Oral cavity	13	32
Oropharynx	12	29
Hypopharinx	5	12
Larynx	11	27
		
Extent of disease		
Recurrent disease		
Locoregional recurrence	14	34
Locoregional and distant recurrence	4	10
Primary disease		
First primary	19	46
Second primary	4	10
		
Prior therapy		
Surgery alone	7	17
Surgery+radiotherapy	6	15
Surgery+radiotherapy+chemotherapy	5	12
Chemotherapy+radiotherapy	4	10
None	19	46

. A total of 18 patients presented a relapse from a previously diagnosed and treated HNSCC, that was locoregional in 14 cases, and both locoregional and distant in four patients (three lung, one skin). Four patients presented a second primary HNSCC, and 19 patients presented with unresectable disease at diagnosis; for the latter two groups, treatment was delivered as induction therapy. Nine patients had received prior chemotherapy; five (56%) had been treated with a combination of cisplatin, 5-FU and paclitaxel as neoadjuvant chemotherapy, and four (44%) had received cisplatin alone as concurrent chemoradiotherapy. In all, 15 patients had received radiotherapy. The median interval between initial diagnosis and relapse or second primary occurrence was 14 months (range 6–53 months).

### Treatment administered

A total of 195 cycles of the study regimen were delivered (median five cycles; range 2–6 per patient): 20 patients (49%) received the six planned cycles, seven (17%) received five cycles, three (7%) received four cycles, six (15%) received three cycles, and five (12%) received two cycles. Most chemotherapy cycles were delivered at the planned doses (94%), and schedules (88%). Doses were reduced in 10 patients (due to seven, one and one episodes of haematologic toxicity, diarrhoea, and hand–foot syndrome (HFS), respectively). Treatment was delayed for a week in 15 patients (due to 13, 1, and 1 episodes of haematologic toxicity, mucositis, and HFS, respectively). Median treatment duration was 16 weeks (range 6–20). Median dose intensities were 25 mg m^−2^ per week of cisplatin (range 15.5–25), and 8.8 g m^−2^ per week of capecitabine (range 5.8–9.3). Median dose intensities achieved for cisplatin and capecitabine were 100 and 95% of the planned doses, respectively.

### Response

In total, 15 responses were documented in the first stage (23 patients), and thus accrual continued. The overall response rate was 68% (28 out of 41 patients; 95% CI, 53–80%) (Table 2

**Table 2:** Response to treatment

	**Patients (*n*=41)**	
	**No.**	**%**
Overall response	28	68
95% CI	53–80	
Complete response	16	39
Partial response	12	29
Stable disease	5	12
Progressive disease	7	17
Nonassessable	1	3

). There were 16 complete responses (39%), 12 partial responses (29%), five disease stabilisations (12%), and seven patients (17%) presented progressive disease as the best response to therapy. One patient (3%) was removed from the study due to cardiac toxicity before response assessment. Median time to response was 9 weeks (range, 8–10 weeks). Median duration of response was 5.6 months (range, 2.4–21 months; 95% CI, 4.2–6.9 months). All patients who ultimately achieved a clinical response were responding by cycle 3, but only 11 out of the 16 complete responses were achieved after three cycles of therapy. No differences were noted comparing response in untreated *vs* previously treated with cisplatin and/or 5FU chemotherapy patients (69 *vs* 67%; *P*=0.91). When analysing the response rate in relapsed subjects according to prior target irradiation, a trend towards a better clinical response rate was documented in nonradiated *vs* radiated targets (85 *vs* 45%; *P*=0.09).

### Toxicity

Haematologic and nonhaematologic toxicities are shown in Table 3

**Table 3:** Summary of haematologic and nonhaematologic toxicity

	**All grades**		**Grade 3–4**	
	**No. of patients**	**%**	**No. of patients**	**%**
*Haematologic*				
Anaemia	29	71	3	7
Neutropenia	17	42	5	12
Thrombocytopenia	6	15	1	2
				
*Nonhaematologic*				
Asthenia	36	88	3	7
Anorexia	34	83	3	7
Hand–foot syndrome	27	66	1	2
Constipation	26	63	0	0
Nausea/vomiting	16	39	0	0
Alopecia	12	29	—	—
Mucositis	11	27	2	5
Neuropathy	4	10	0	0
Diarrhoea	3	7	1	2
Cardiotoxicity	1	2	1	2

. Grade 3–4 neutropenia was documented in five subjects, but no febrile neutropenic episodes were recorded. Median nadir ANC count was 2.2 × 10^9^ l^−1^ (range, 0.34–11.2 × 10^9^ l^−1^). Thrombocytopenia was observed in six patients (15%), that was grade 3 in one subject (2%). Anaemia was documented in 29 patients (71%), and was grade 3 in three subjects (7%). Asthenia, anorexia, HFS, and constipation were the most frequent nonhaematologic events. Nine out of 41 patients (22%) suffered from grade 3 or 4 nonhaematologic adverse events, being the most frequent asthenia and anorexia (three grade 3 episodes each), and mucositis (two grade 3 events); the latter was unrelated to prior radiotherapy. Only one patient suffered from grade 3 HFS. One 47-year-old woman with a past history of smoking presented an episode of exercise-related angor pectoris on day 12 of the second cycle of therapy that responded to calcium-channel blockers, and was thought related to capecitabine administration; subsequently, she was removed from the study. Table 4

**Table 4:** Toxicity by treatment cycle

**Cycle**	**1 (*n*=41)**				**2 (*n*=40)**				**3 (*n*=36)**				**4 (*n*=30)**				**5 (*n*=27)**				**6 (*n*=20)**			
**Grade**	**1**	**2**	**3**	**4**	**1**	**2**	**3**	**4**	**1**	**2**	**3**	**4**	**1**	**2**	**3**	**4**	**1**	**2**	**3**	**4**	**1**	**2**	**3**	**4**
Anaemia	6	1	0	0	11	1	1	0	7	2	1	0	12	3	0	0	7	6	1	0	9	2	1	0
Neutropenia	2	1	0	1	2	0	2	1	4	1	1	0	2	2	0	0	2	1	1	0	3	0	0	0
Thrombopenia	1	0	0	0	2	0	0	0	1	0	0	1	1	2	0	0	1	0	0	1	0	1	0	0
Asthenia	16	8	0	0	18	9	1	0	18	8	1	0	15	8	1	0	14	8	2	0	10	8	0	0
Anorexia	8	3	0	0	11	8	0	0	16	6	1	0	11	7	1	0	14	8	2	0	11	6	0	0
Hand–foot	0	0	0	0	0	1	0	0	8	2	0	0	16	3	1	0	8	10	1	0	6	11	0	0
Constipation	11	2	0	0	12	4	0	0	9	3	0	0	12	1	0	0	11	2	0	0	6	3	0	0
Nausea/vomiting	6	1	0	0	5	0	0	0	4	0	0	0	2	3	0	0	2	0	0	0	1	1	0	0
Alopecia	0	0	—	—	2	0	—	—	9	0	—	—	8	0	—	—	8	1	—	—	7	0	—	—
Mucositis	3	1	2	0	2	2	1	0	2	1	0	0	2	0	0	0	1	0	0	0	0	0	0	0
Neuropathy	0	0	0	0	0	0	0	0	1	0	0	0	2	0	0	0	0	1	0	0	2	1	0	0
Diarrhoea	0	0	1	0	0	0	0	0	1	0	0	0	1	0	0	0	0	0	0	0	0	0	0	0
Cardiotoxicity	0	0	0	0	0	0	1	0	0	0	0	0	0	0	0	0	0	0	0	0	0	0	0	0

shows toxicity by treatment cycle.

### Subsequent therapy and outcome

After completion of the study treatment, patients with disease control or unassessable were treated with radiotherapy (12), surgery plus radiotherapy (five), surgery (four), and 13 received no further therapy. Patients with progressive disease as the best response were treated with chemotherapy (four), chemotherapy and radiotherapy (two), and best supportive care (one).

**Figure 1:**
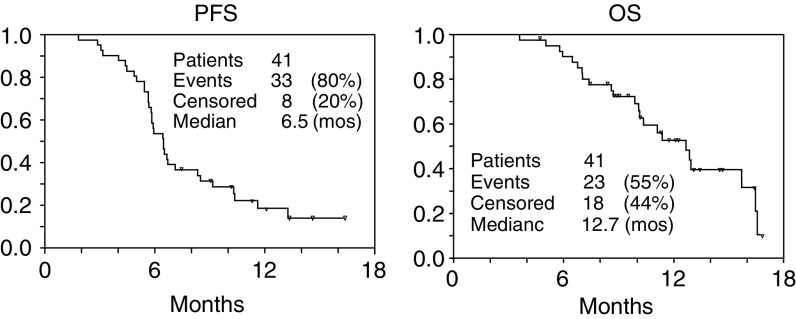
Progression-free survival and OS for the whole series of patients.

In all, 33 patients have progressed; 27 patients recurred locoregionally, two patients presented distant metastases, and four patients presented both locoregional and distant disease. Median PFS was 6.4 months (95% CI, 5.7–7.3 months), and median survival after disease progression was 3 months (95% CI, 0.7–5.3 months). A total of 23 deaths have occurred, all of them related to tumour progression. Median overall survival was 12.6 months (95% CI, 10.0–15.2 months) (Figure 1

). The multivariate model showed that achieving a complete response marginally predicted a better OS (*P*=0.041).

### Comparison of patients with recurrent and primary unresectable disease

Results are summarised in Table 5

**Table 5:** Comparison between patients with recurrent and primary unresectable disease

	**Recurrent disease (*n*=18)**		**Primary disease (*n*=23)**	
	**No.**	**%**	**No.**	**%**
*Response*				
Overall response	11	61	17	74
95% CI	38–80		53–87	
Complete response	6	33	10	44
Partial response	5	28	7	30
Stable disease	1	6	4	17
Progressive disease	5	28	2	9
Nonassessable	1	6	0	0
				
*Further therapy*				
Radiotherapy	2	11	10	44
Surgery	2	11	2	9
Surgery plus radiotherapy	1	6	4	17
Chemotherapy	4	22	0	0
Chemotherapy plus radiotherapy	0	0	2	9
None	9	50	5	22
				
*Relapses*				
No relapse	1	6	7	30
Locoregional	12	67	15	65
Distant	1	6	1	4
Locoregional plus distant	4	22	0	0

**Figure 2:**
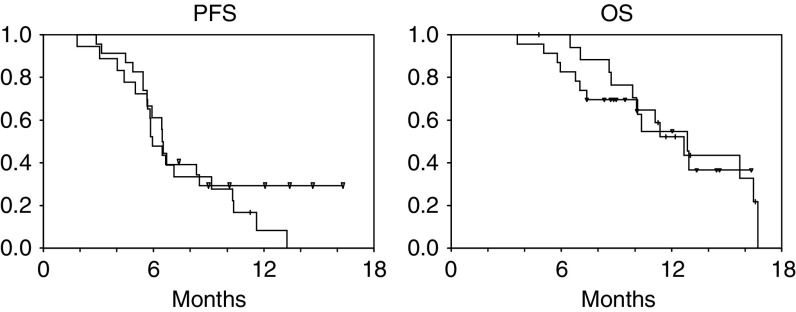
Comparison of PFS and OS in recurrent (+) *vs* primary unresectable (∇) patients.

. Response rates were similar between patients with recurrent disease and those with primary unresectable disease (61 *vs* 74%, respectively; *P*=0.50), although there was a trend towards a higher incidence of progressive disease in the recurrent group (9 *vs* 28%, *P*=0.10). Toxicity was equivalent between groups. PFS (6.5 *vs* 5.9 months, *P*=0.40) and OS (12.7 *vs* 12.9 months, *P*=0.73) showed no differences when comparing patients with recurrent and primary unresectable disease, respectively (Figure 2

). However, the proportion of patients free of relapse at the moment of this analysis was marginally higher in patients with primary tumours (6 *vs* 30%, *P*=0.046).

### Immunohistochemical evaluation

Tumour tissue from 31 patients was assessed. The levels of expression of TS, TP-cyt, TP-nuc, and VEGFR1 to 3 are shown in Table 6

**Table 6:** Level of expression of TS, TP-cyt, TP-nuc, and VEGFR 1–3

	**No expression (%)**	**Low expression (%)**	**High expression (%)**
TS	7 (23)	14 (47)	9 (30)
TP-cyt	7 (23)	13 (42)	11 (35)
TP-nuc	13 (42)	13 (42)	5 (16)
VEGFR 1	18 (58)	13 (42)	0 (0)
VEGFR 2	1 (3)	4 (13)	26 (84)
VEGFR 3	1 (3)	27 (87)	3 (9)

TP-cyt: cytoplasmic thymidine phosphorylase; TP-nuc: nuclear thymidine phosphorylase; TS: thymidine synthase; VEGFR: vascular endothelial growth factor receptor.

. The only factor that showed a significant association with any clinical or outcome parameters was TP-cyt (Table 7

**Table 7:** Correlation of the levels of expression of cytoplasmic thymidine phosphorylase (TP-cyt) with clinical features

	**TP-cyt**		
	**No or low expression (%)**	**High expression (%)**	***P***
*Primary tumour site*			
Oral cavity	6 (55)	5 (45)	
Oropharynx	9 (90)	1 (10)	0.009
Hypopharinx	4 (100)	0 (0)	
Larynx	1 (17)	5 (83)	
			
*Type of disease*			
Recurrence	5 (36)	9 (64)	0.007
Primary	15 (88)	2 (12)	

TP-cyt: cytoplasmic thymidine phosphorylase.

). High TP-cyt expression was more prevalent in patients with a laryngeal location than in those with neoplasms arising from other locations (83 *vs* 24%; *P*=0.009). In addition, a higher percentage of patients with a recurrence expressed high levels of TP-cyt when compared with those with primary disease (64 *vs* 12%; *P*=0.007). No significant correlation was found between TP-cyt and TP-nuc (Pearson, *r*=0.23; *P*=0.22). No other differences were found. MVD count was higher in patients with a recurrence than in those with primary disease (31±7 *vs* 23±6 MPF; *P*=0.012). There were no differences in MVD when stratifying patients according to clinical characteristics, or the expression of other histologic factors. No significant correlation was found between MVD and TP-cyt (Pearson, *r*=0.28; *P*=0.15), or TP-nuc (Pearson, *r*=−0.15; *P*=0.47). Patients with an MVD above the median (23 MPF) showed a trend towards a longer OS compared to those with an MVD below the median (12.9 *vs* 10.1 months; *P*=0.074).

## DISCUSSION

The combination of cisplatin and capecitabine at the doses evaluated showed a significant level of activity, and a good tolerability. The median number of cycles administered and the dose intensity achieved highlight the feasibility of this regimen. Although it was not the purpose of this trial, and with due caution considering the limited sample size, it is not adventurous to affirm that this regimen shows at least a similar level of activity as that of cisplatin and infusional 5FU. The relative heterogeneity of this cohort of patients permitted to establish a number of thought-provoking comparisons. No difference in terms of efficacy was observed between patients with a relapse and those with primary disease, nor was any dissimilarity evidenced between cisplatin and/or 5FU-treated and previously untreated subjects. Also, the response rate according with prior target irradiation was nonsignificantly higher in nonradiated patients. However, considering the low number of patients analysed, these observations have to be interpreted with caution.

Interestingly, all patients who ultimately responded had significant tumour shrinkage after three cycles, an observation that emphasises the activity of the regimen; notwithstanding, five out of 16 complete responders needed four or more cycles to achieve a maximal effect. This observation may be relevant in decision-making strategies, for it may indicate that only those patients responding by cycle three will benefit from further therapy.

Toxicity was moderate and manageable; anaemia was the main haematologic toxicity and was predominantly grade 1. Neutropenia was documented in five patients but none of them developed related infectious complications. This profile of haematological toxicity is lower than that consistently reported with cisplatin–5FU (Forastiere *et al*, 2003). The most common nonhaematologic toxicities were asthenia, anorexia, HFS, and constipation. Of note, a patient developed angor pectoris while receiving capecitabine, an event that highlights the pharmacologic similarities of this drug to infusional 5FU. The toxicity profile appears to be milder to that reported by Pivot *et al* (2003), even considering that the average number of cycles per patient is higher in the current study (2.5 *vs* 4.8 cycles per patient (median not reported)). Also efficacy in the present study was higher. Differences in patient population may explain these findings; in fact, all patients enrolled in that phase I study had been previously treated with chemotherapy, as opposed to only 22% of patients in this study. However, the higher dose of cisplatin used (17 out of 21 patients received 100 mg m^−2^) may be partially responsible for the increased incidence of adverse events; in view of the efficacy of the current schedule and the relative equivalency of lower *vs* higher doses of cisplatin in advanced HNSCC patients (Veronesi *et al*, 1985), 75 mg m^−2^ seems to be a reasonable cisplatin dose to administer in combination with capecitabine.

As long as it maintains equivalent efficacy, patients generally prefer oral administration of chemotherapy to intravenous infusions (Liu *et al*, 1997), but oral therapy may theoretically be difficult in patients with HNSCC who frequently have swallowing dysfunction related either to their tumour or its treatment. However, this was not the case in the present report, a fact underlined by the high compliance with oral chemotherapy documented (98%).

Tumour angiogenesis manifested as increased MVD counts or VEGF expression has been shown to adversely impact outcome and treatment results in HNSCC patients in several series (O-Charoenrat *et al*, 2001; Mineta *et al*, 2002). After analysing 94 patients with unresectable HNSC treated with chemoradiation, Koukourakis *et al* (2000) concluded that TP-nuc expression was associated with angiogenesis as assessed by MVD, with resistance to radiotherapy and cytotoxic therapy, and with poorer survival in squamous cell head and neck cancer. TP is a pertinent factor to be assessed in this clinical context given its unique duality: it is both the activator of one of the drugs administered (and thus with potential predictive capability), and a potent proangiogenic factor. Recently, Brown *et al* (2000) showed that TP induces oxidative stress to cancer cells, and promotes secretion of angiogenic factors such as VEGF. In the present report, TP-cyt expression was found to be significantly more prevalent in patients with laryngeal cancer compared with other locations, and in relapsed patients. The significance of the former finding remains obscure, whereas the latter observation may be related to the aggressiveness of the disease. The second potential predictive factor of response assessed was TS. A previous study shows that low intratumoral expression of TS was a strong predictor of response to 5FU-based therapy of disseminated colorectal cancers, and strongly correlated with a longer survival (Leichman *et al*, 1997). However, no relationship was found between TP (either nuclear or cytoplasmatic) or TS and activity in the current series. The levels of the VEGFR are correlated with a poorer grade of tumour differentiation and prognosis in pancreatic cancer (Buchler *et al*, 2002). The fact that in the current series the majority of patients expressed high levels of VEGFR2 but none expressed high levels of VEGFR1, together with the lack of correlation with MVD, suggests that the sensitivity and/or specificity of the technique may not be optimal. However, and this also applies to TP/TS expression, the interpretation of these results is constrained by the relatively low number of patients analysed.

MVD count was significantly higher in relapsed patients than in those with primary disease, and it could be hypothesised that a higher MVD in recurrences indicates a more aggressive behaviour, but the present study is underpowered to address this issue. The fact that both MVD and TP-nuc were concomitantly elevated in relapsed patients may indirectly confirm the association between these two factors in prior reports (Koukourakis *et al*, 2000). It is also somehow paradoxical that patients with an MVD above the median showed a trend towards a better outcome; however, the multivariate analysis showed no relationship between MVD (either as a continuous value of grouping according to the median) and OS. It is increasingly evident that univariate results from studies involving analysis of biologic factors have to be cautiously interpreted, considering the numerous stratifications and comparisons performed.

In summary, this cisplatin–capecitabine schedule in recurrent or unresectable HNSCC is feasible and effective in terms of tumour response, and comparative, multicentre trials with cisplatin–5FU seem warranted.
